# Laparoscopic Hemostasis of Intractable Delayed Postpartum Hemorrhage

**DOI:** 10.7759/cureus.12892

**Published:** 2021-01-25

**Authors:** Dipak Limbachiya, Mangirish Kenkre, Siddharth Shah, Rashmi Kumari, Hardik D Desai

**Affiliations:** 1 Department of Gynecology, Eva Women's Hospital, Ahmedabad, IND; 2 Internal Medicine, Gujarat Adani Institute of Medical Sciences, Bhuj, IND

**Keywords:** postpartum hemorrhage, postpartum bleeding, laparoscopy, laparoscopic surgery, hemostasis

## Abstract

Postpartum hemorrhage (PPH) is associated with considerable morbidity and mortality, particularly when relaparotomy is necessary. The etiology of spontaneous intractable PPH in a hemodynamically stable patient is poorly understood and remains open to speculation. Secondary, or delayed, PPH is usually defined as the excessive bleeding from the genital tract, with a loss of 500 ml or more of blood occurring after the first 24 hours after delivery until the sixth week of puerperium. In this report, we present three cases of severe, diffuse postpartum bleeding unresponsive to conventional hemostatic measures, which were successfully managed laparoscopically at our center. In all three cases, hemostasis was accomplished by using a laparoscopic procedure: with the excision of cervical stump bleeding in the first case, bilateral uterine artery ligation accompanied by laparoscopic hysterectomy in the second case, and bilateral internal iliac artery ligation in the third case.

## Introduction

Postpartum hemorrhage (PPH) can lead to considerable morbidity and mortality, particularly in cases where relaparotomy is required. The etiology of spontaneous intractable PPH in a hemodynamically stable patient is not fully understood. Secondary, or delayed, PPH is characterized by excessive bleeding from the genital tract and has been associated with the loss of 500 ml or more of blood occurring after the first 24 hours after delivery until the sixth week of puerperium [1-2]. Vigilance is essential in recognizing ongoing bleeding in the postpartum period while prompt and rapid action is needed to achieve control of unanticipated postpartum bleeding. When severe intraoperative coagulopathy or hemodynamic instability is present, the damage control sequence forms part of a valuable surgical strategy. Such maintenance of surgical hemostasis in intractable cases of PPH can be challenging for even the most technically talented surgeon.

Laparoscopy was introduced in the late 1980s. It has proven to be a credible therapeutic intervention and has been credited with heralding a new surgical age. It has greatly contributed to our management of surgical emergencies. However, the evidence for the use of therapeutic laparoscopy in literature remains sparse, with the majority of the publications being case reports pertaining to laparoscopic repair of perforating injuries to the diaphragm [3-5], laparoscopic hemostasis of minor injuries to the liver or spleen [6-7], and repair of limited gastrointestinal injuries [8]. In gynecology, the evidence for the use of laparoscopy is limited to the management of ectopic pregnancy [9], ovarian cyst torsion [10], and salpingo-oophoritis [11]. We hereby report three cases of severe, diffuse postpartum bleeding unresponsive to conventional hemostatic measures. The patients were successfully managed laparoscopically at our center. The minimally invasive nature of laparoscopic surgery, coupled with fast postoperative recovery, presents an efficient and patient-friendly option for many laparoscopic surgeons when managing hemodynamically stable patients with PPH. In line with other studies in the literature, our case series of three cases show that laparoscopic intervention is a feasible option for intractable cases of postpartum bleeding.

## Case presentation

Case 1

A 28-year-old primigravida of Indian nationality with 35 weeks of gestation, married for five years, with in vitro fertilization (IVF) conception of twin gestation presented to our tertiary care center with the complaint of premature rupture of membranes. Antenatally, the patient had developed preeclampsia at 32 weeks of gestation with a urine albumin level of 1+. She was started on labetalol 100 mg twice a day. She had also received a prophylactic injection of betamethasone at 32 weeks. On examination, her pulse rate was 90/minute, BP was 140/90 mmHg, and urine albumin was 1+. Per speculum examination was suggestive of frank leaking, and per vaginal (PV) examination revealed a Bishop score of 5 with the presentation of the first fetus as breech. Hence, the decision for an emergency lower segment cesarian section (LSCS) was taken. LSCS was performed with a Pfannenstiel incision on the skin. Surgery was uneventful, and two babies weighing 2 kg and 1.8 kg were delivered.

Four hours after the LSCS, the patient developed atonic PPH. Medical management, uterine massage, and uterine packing were attempted, but the bleeding could not be controlled. A bleeding disorder was ruled out. The decision for exploratory laparotomy with midline vertical incision was taken. On laparotomy, the uterus was atonic, the cesarean scar was intact, and no localized hematoma or hemoperitoneum was seen. Hence, bilateral uterine and ovarian arteries were ligated, followed by hemostatic sutures on the uterus. But despite these measures, bleeding still could not be controlled. At this stage, resuscitative measures were initiated. The decision for hysterectomy was taken and subtotal obstetric hysterectomy was performed. Subsequently, hemostasis was achieved, an intraperitoneal drain was placed, and the patient was kept in a high-dependency unit.

Two hours after laparotomy, the patient's condition deteriorated again. On per speculum examination, bleeding from the cervical stump was seen. Vaginal packing was done. After one hour, packing was shocked, and again packing was changed but the bleeding did not stop. At that time, her pulse rate was 165/minute, BP was not recordable, and the monitor was not showing oxygen saturation (SpO_2_). The drain output was 5 ml. The patient was kept on inotropic support with continuous blood transfusion and proper fluid management.

Eventually, the decision was taken for re-exploration by laparoscopic approach. Pneumoperitoneum was created through drain tube access. The intraoperative laparoscopic finding revealed bleeding from the cervical stump with parametrial hematoma. Retroperitoneal dissection was performed. Following this, bilateral internal iliac arteries were identified and Hem-o-Lok® clips (Weck Closure Systems, Research Triangle Park, NC) were applied. Ureterolysis was done and the parametrial hematoma was drained. Bilateral parametria were dissected. The bleeding cervical stump along with parametria was dissected and excised. The vault was closed with Vicryl No. 1 suture (Ethicon, Inc., Somerville, NJ). Hemostasis was achieved, and a total of six units of blood with eight fresh frozen plasma and six platelet-rich plasma were transfused, and the patient was gradually weaned off from inotropic supports. The patient was kept in the ICU under observation for 48 hours. She was then shifted to the ward with stable vitals and discharged on postoperative day five.

**Figure 1:**
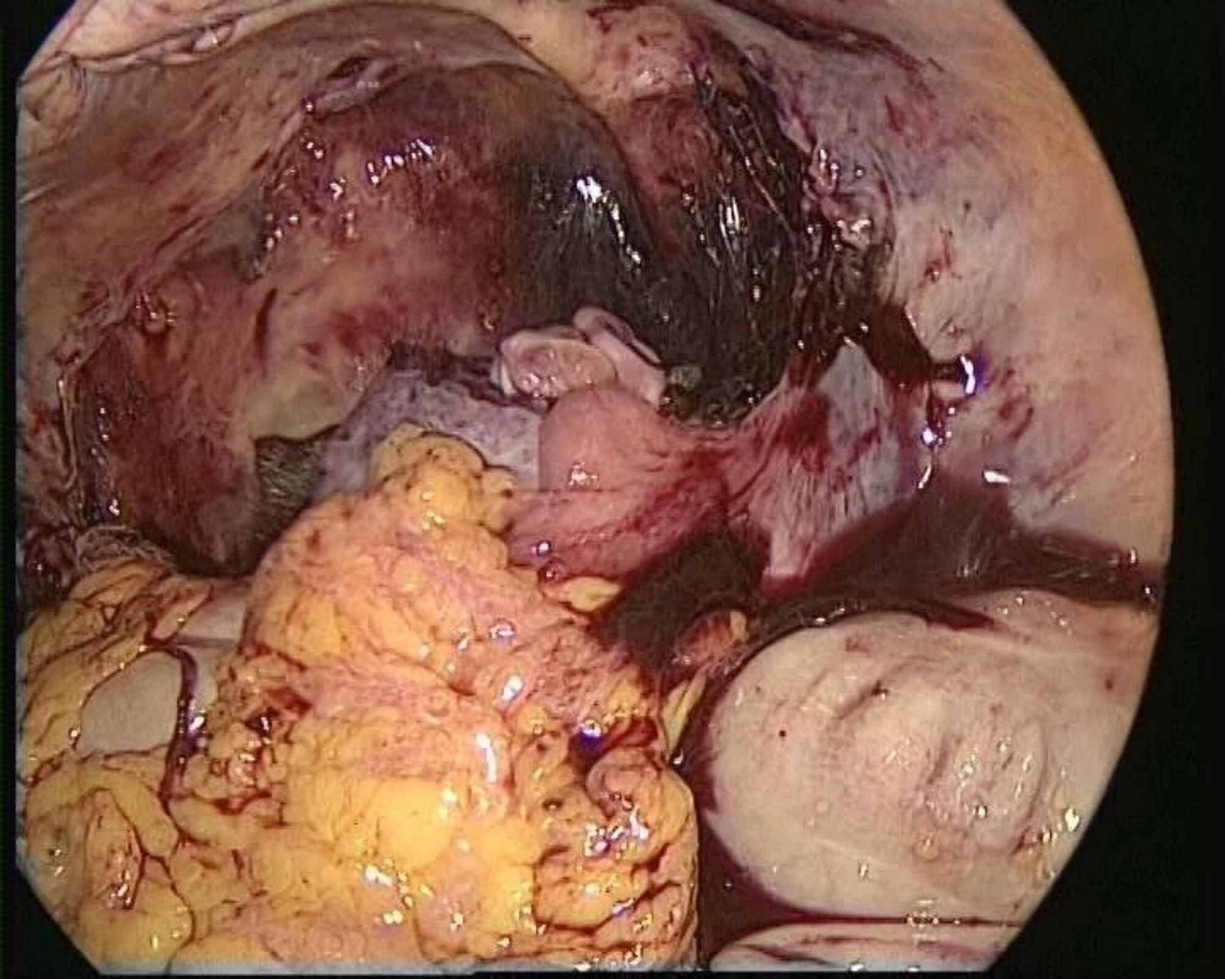
First endoscopic view of case 1

**Figure 2:**
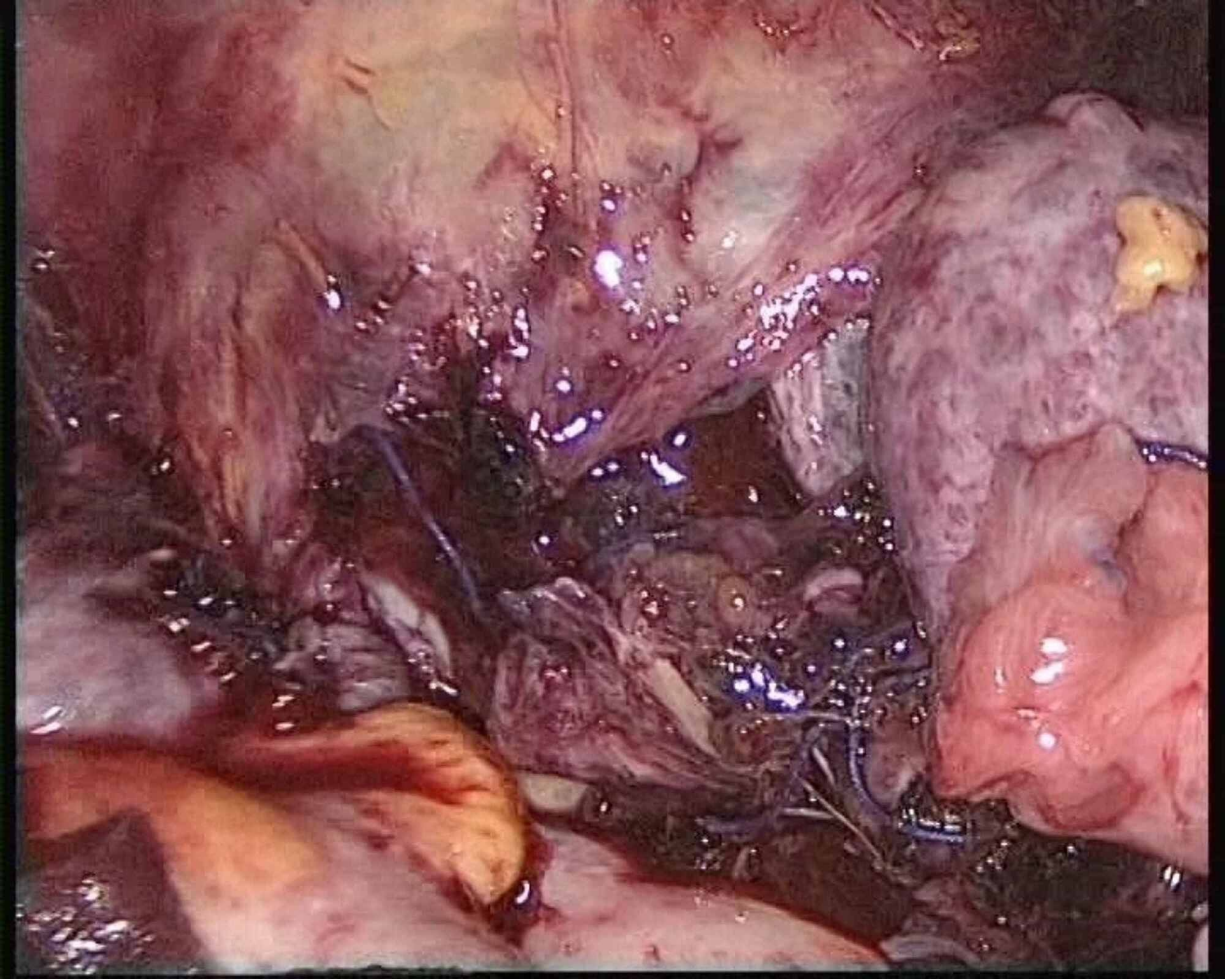
Bleeding from the cervical stump in case 1

**Figure 3:**
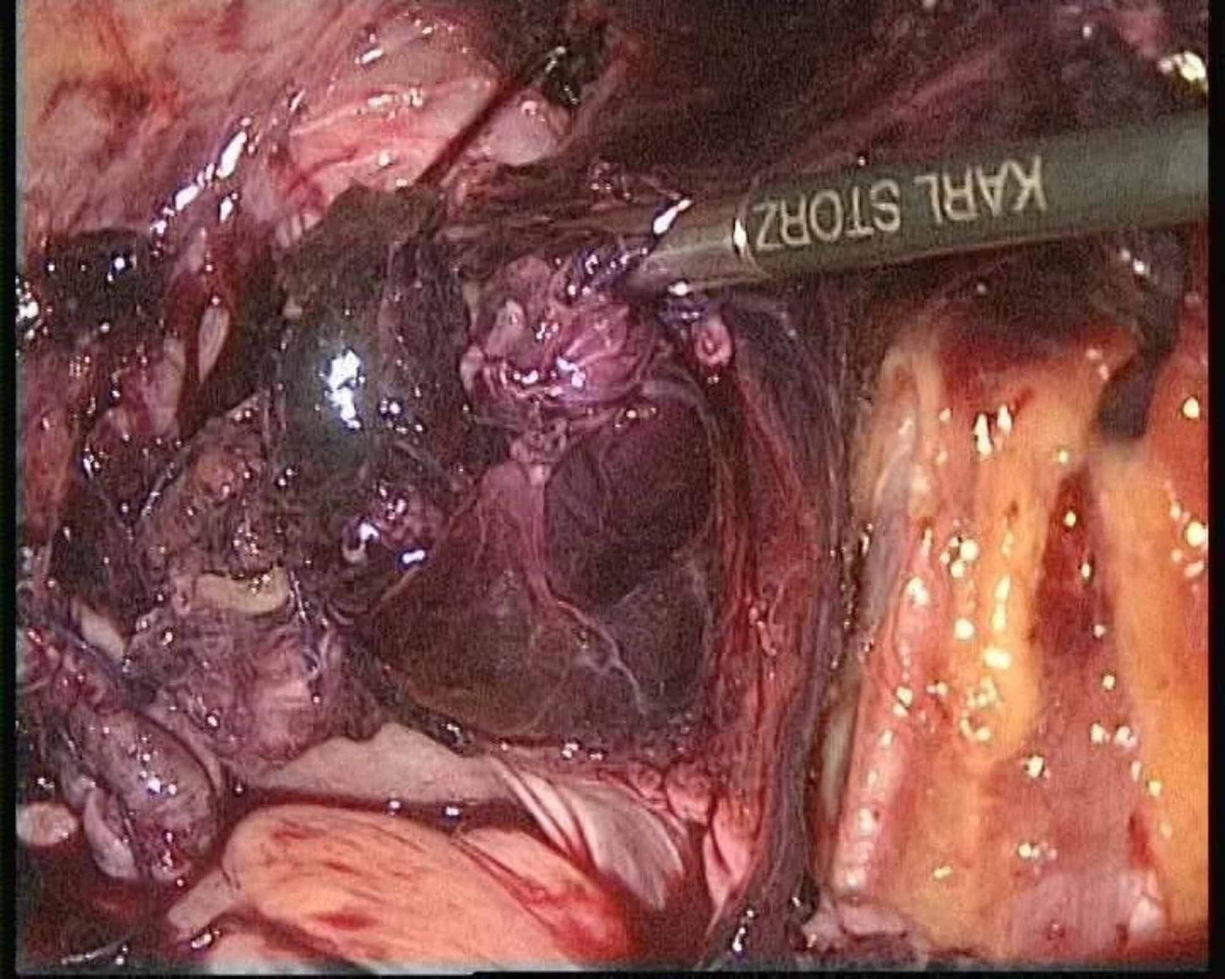
Parametrial hematoma seen in case 1

**Figure 4:**
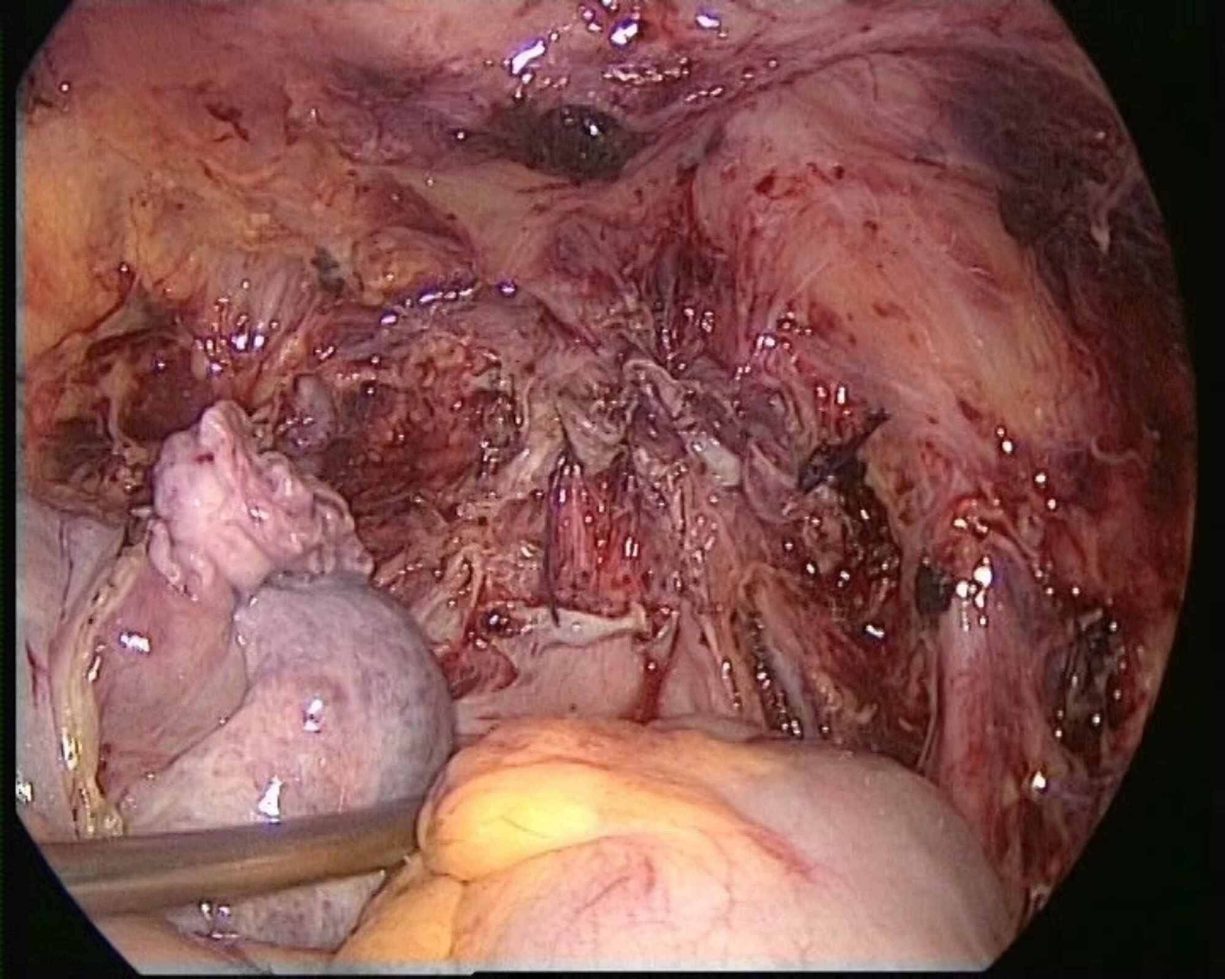
Final endoscopic view of case 1

In addition to transvaginal sonography, transabdominal sonography also showed an empty uterine cavity and empty cervical canal with a gestational sac in the anterior myometrium of the lower uterine portion (Figure 1, Figure 2, Figure 3, Figure 4).

Case 2

A 26-year-old woman presented for a routine antenatal check-up at our hospital, after two months of amenorrhea and a positive urine pregnancy test. She was otherwise asymptomatic. She had a history of two previous cesarean sections: the first one performed four years back for intrapartum fetal distress and the second one performed two-and-half years back due to scar tenderness.

**Figure 5:**
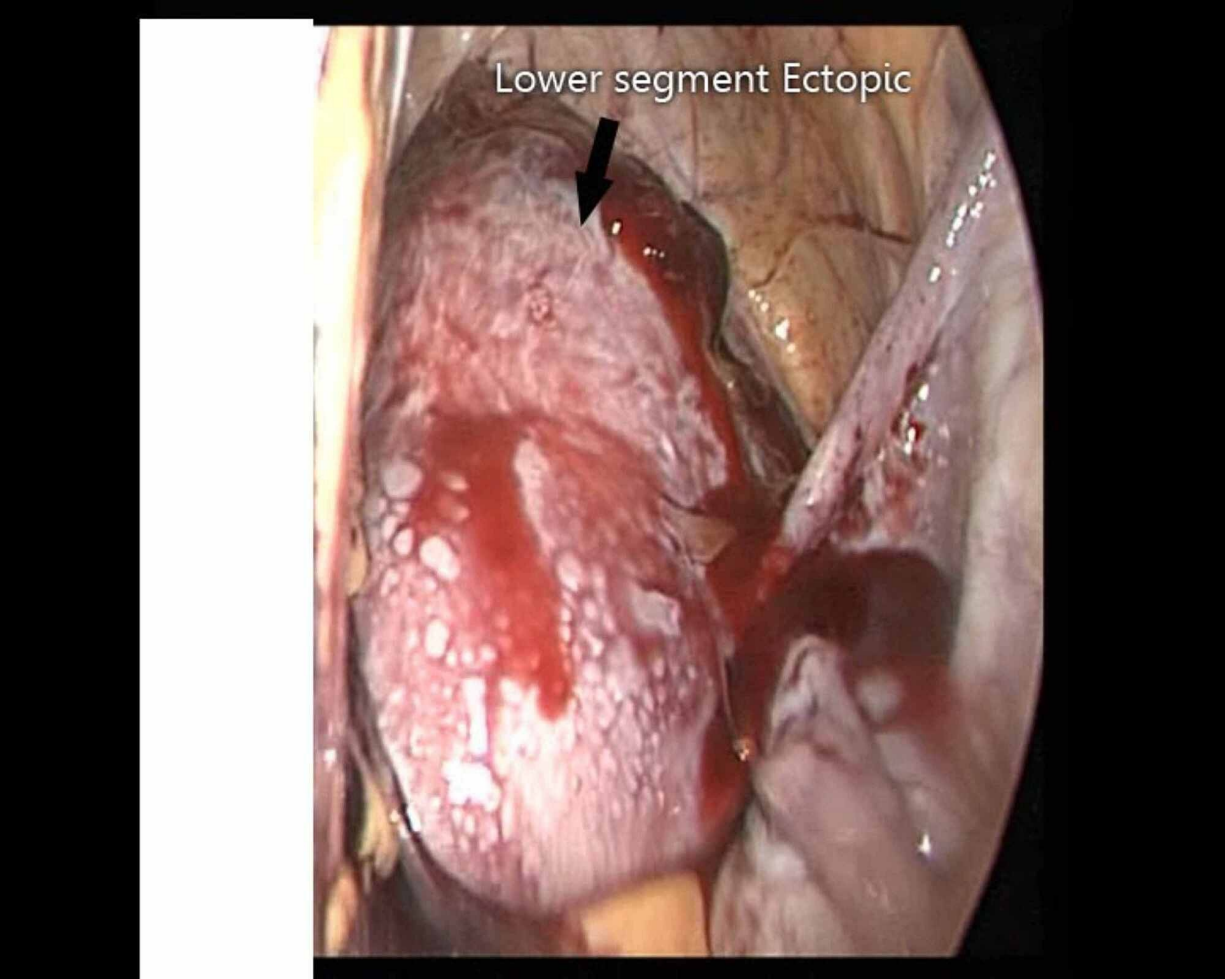
Lower segment ectopic scar in case 2

She was advised to undergo routine first-trimester sonography. The gestational sac had a fetal pole and yolk sac within, showing fetal cardiac activity and having an average gestational age of six weeks and six days. Posteriorly, the gestational sac was seen extending into the endometrial cavity in the lower uterine segment while the anterior myometrium anterior to the gestational sac was thinned out. The patient reported to the emergency department at 14 weeks of gestation with complaints of severe pain in the abdomen and dizziness over the last six hours. Clinical examination revealed the following findings - pallor (3+), pulse rate: 90/minute, BP: 80/50 mmHg. The decision for emergency laparoscopy surgery was taken. Perioperative findings can be seen in Figure 5.

**Figure 6:**
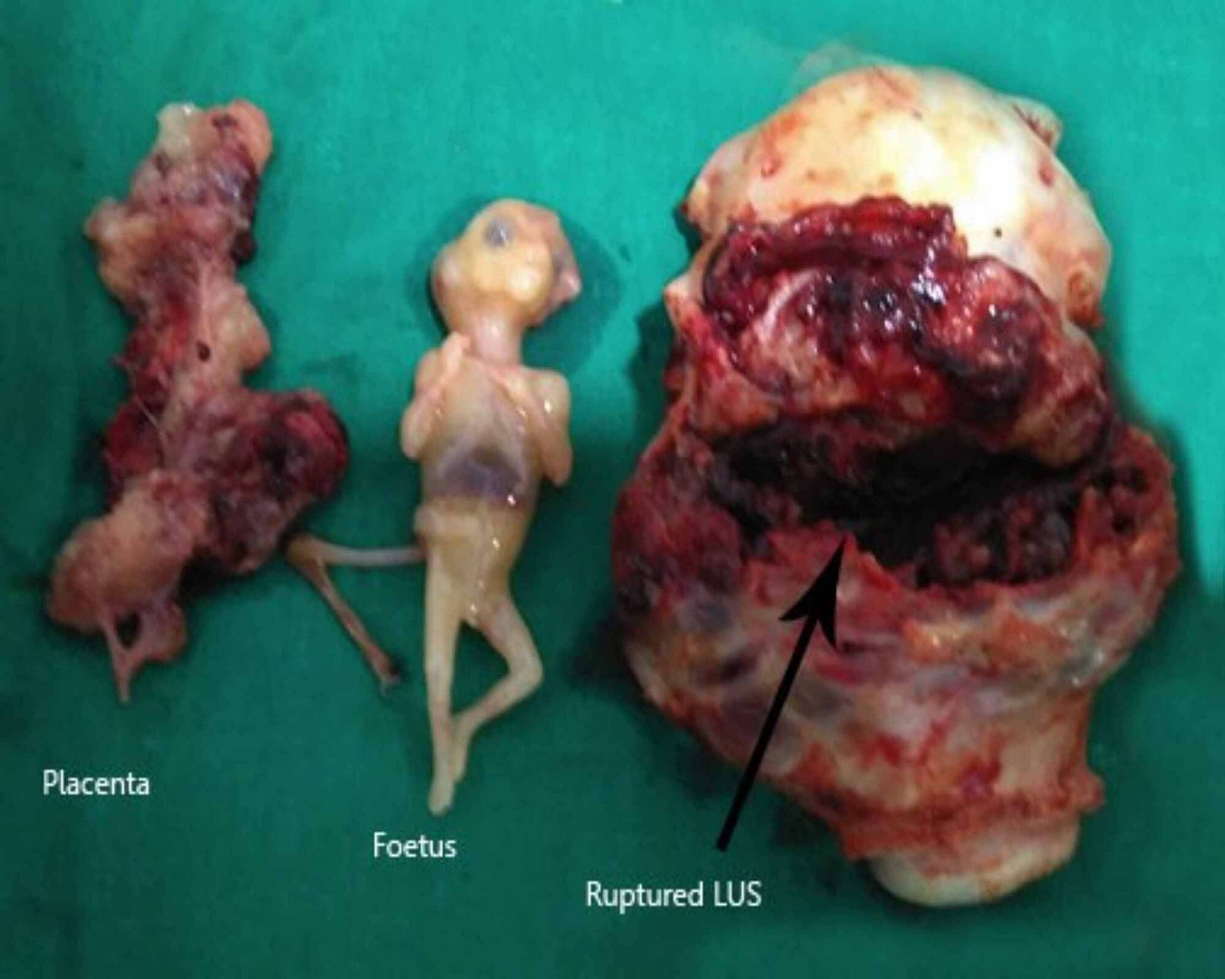
Excised placenta, fetus, and ruptured LUS in case 2 LUS: lower uterine segment

Hemoperitoneum associated with clots was present (approximately 2.5-3 liters). There was a full-thickness, full-length scar rupture at the previous uterine scar site, with bulging of placental tissues into the peritoneal cavity. Immediately, bilateral uterine arteries were ligated at their origin. The fetus and placental tissues were removed from the uterus and the exact size and site of uterine rupture were reassessed. It was a full-thickness and a full-length scar rupture, extending almost from one to the other pelvic wall. Since the patient already had two living issues and owing to the continuous bleeding from the placental bed, the decision for laparoscopic hysterectomy was taken. Intraoperatively, the patient received three pints of packed cell volume (PCV) and two pints of Haemaccel (Piramal Healthcare, Mumbai, India). Postoperatively, two more pints of PCV were given. She tolerated the procedure well. The patient was discharged on postoperative day three in a stable condition (Figure 6).

Case 3

A 28-year-old female [para 2, living 2 (P2 L2)], had undergone an elective cesarean one month prior to her presentation in view of a previous cesarean section. The surgery had been uneventful, and she had been discharged on the sixth postoperative day. She came to the OPD after seven days with the complaint of bleeding per vaginum with a history of the passage of clots. On examination, bleeding was found to be more than average. The patient was medically managed, and the subsequent ultrasonography returned normal findings.

After transfusing one unit of blood and giving antibiotic coverage, the patient was discharged with bleeding per vaginum within the normal limit. The patient presented to the OPD again after 10 days with the same complaints. On PV examination, bleeding was still present. Intrauterine packing was done and kept for 24 hours. Packing was removed after 24 hours, and she was kept under observation for the next 48 hours with no bleeding PV. Again, after 10 days, the patient returned with the same complaint with ultrasound suggestive of a focal hypogenic mass devoid of any vascularity extending from endometrium to subserosa on the left side. The patient was referred to our center and the decision was taken to manage the case laparoscopically.

Intraoperative findings were as follows: bladder was found adhered to the anterior uterine wall, which was dissected down. A Hematoma was present at the right end of the cesarean scar, extending up to the broad ligament. Cesarean scar sutures had given away. Active bleeding was present from the right angle of the scar.

Retroperitoneal dissection was performed, and bilateral internal iliac arteries were ligated. The cesarean scar site was exposed, and older suture material was removed. Complete scar site was restored with Vicryl No. 1 after taking both angles separately. Hemostasis was confirmed. The patient had an uneventful recovery and was discharged on the day after surgery. Ultrasound performed at eight weeks postpartum showed a normal uterus.

## Discussion

The management of delayed intractable PPH requires a step-wise escalation of pharmacological and surgical approaches. Delayed intractable PPH in young women or women with low parity poses a surgical dilemma for obstetricians. The treatment options for PPH include conservative management with uterotonic drugs, external compression with uterine sutures (B-Lynch, Hayman, Cho), selective devascularization by ligation or embolization of the uterine artery, and intrauterine packing [12-16]. The choice of treatment depends on several factors: delivery mode, the volume of bleeding, the site of origin, the patient's hemodynamic tolerance, and certain human and technical factors. When PPH persists despite conservative management and aggressive medical treatment, prompt consideration should be given for surgical intervention. These include vascular occlusive methods like internal iliac artery ligation, uterine compression sutures (B-Lynch), uterine balloon tamponade, and peripartum hysterectomy to control the life-threatening hemorrhage [17]. To our knowledge, the laparoscopic approach to internal iliac artery ligation has not been reported from the Indian standpoint. We successfully achieved hemostasis in these three cases by utilizing a different, novel, effective, and minimally invasive surgical technique in intractable delayed PPH. Fertility-preserving procedures are encouraged, especially in young women. In our case series, all three females were aged between 26 and 28 years. Hysterectomy proved necessary in two cases due to the intractable nature of bleeding. One of the cases had two living issues, which reinforced our decision to perform a hysterectomy. Timely decision-making and intervention can help avoid consumption coagulopathy and further deleterious consequences.

A few other studies have reported laparoscopic approaches to check PPH. Chou et al. [18] described the case of a 29-year-old woman with delayed PPH in whom curettage of the uterine cavity to remove retained placenta was performed; however, the bleeding did not stop. They further opted for laparoscopic bipolar coagulation of uterine vessels and successfully managed to arrest bleeding and preserve the uterus. Similarly, Panuccio et al. [19] also described a rare case of laparoscopic coagulation of hypogastric artery technique in a patient with PPH. When performing a laparoscopic abdominal re-exploration for PPH, the surgeon should be mindful of the various patterns in which PPH bleeding may occur. In our three cases, bleeding was from the cervical stump and the previous LSCS scar rupture. Usually, discreet bleeding sites are easier to detect and manage. This form of postoperative hemorrhage typically results from inadequate hemostasis at a previous repair site. Due to disease-induced vessel wall rigidity, bleeding from a specific vessel or group of vessels may result from vascular injury during suture ligation, suture displacement from a short vascular "stump," or insufficient vascular occlusion by a suture [20]. The early identification of this potentially devastating complication is perhaps the most difficult part of postoperative hemorrhage management. The surgeon must stay alert for signs of continuing hemorrhage during the postoperative period. Subtle clinical signs of blood loss may be overlooked or wrongly attributed to postoperative fluid shifts, especially when there are no serious bleeding problems during the initial operation. In our three cases, the management team was monitoring pulse, BP, SpO_2,_ and drain output, which guided the treatment decision. The decision to return to the operating room should rely on whether the patient's anticipated postoperative blood loss exceeds the standards of the operative surgeon.

## Conclusions

A hemorrhage is a potentially catastrophic complication of obstetric surgery. In order to recognize the clinical symptoms relevant to postoperative hemorrhage, the operating surgeon should be very alert. A meticulous surgical technique is important for its management. Prompt surgical action is crucial for the patient's survival. Using the laparoscopic approach requires the service of surgeons experienced in advanced laparoscopy, along with appropriate equipment and support. The means to convert to laparotomy should always be available because of the risk of uncontrolled bleeding. To complete the process with optimal results, a disciplined and organized approach is required.
